# Dynamic Context-Aware Event Recognition Based on Markov Logic Networks

**DOI:** 10.3390/s17030491

**Published:** 2017-03-02

**Authors:** Fagui Liu, Dacheng Deng, Ping Li

**Affiliations:** School of Computer Science and Engineering, South China University of Technology, Guangzhou 510006, China; fgliu@scut.edu.cn (F.L.); 18813759473@163.com (P.L.)

**Keywords:** event recognition, sensing data, information fusion, Markov logic networks, dynamic uncertainty

## Abstract

Event recognition in smart spaces is an important and challenging task. Most existing approaches for event recognition purely employ either logical methods that do not handle uncertainty, or probabilistic methods that can hardly manage the representation of structured information. To overcome these limitations, especially in the situation where the uncertainty of sensing data is dynamically changing over the time, we propose a multi-level information fusion model for sensing data and contextual information, and also present a corresponding method to handle uncertainty for event recognition based on Markov logic networks (MLNs) which combine the expressivity of first order logic (FOL) and the uncertainty disposal of probabilistic graphical models (PGMs). Then we put forward an algorithm for updating formula weights in MLNs to deal with data dynamics. Experiments on two datasets from different scenarios are conducted to evaluate the proposed approach. The results show that our approach (*i*) provides an effective way to recognize events by using the fusion of uncertain data and contextual information based on MLNs and (*ii*) outperforms the original MLNs-based method in dealing with dynamic data.

## 1. Introduction

Event recognition is the process of automatically identifying interesting status changes of entities or physical environments. With the advancement of context-aware applications in smart spaces, event recognition is regarded as a sound method to offer a constantly changing situational picture about observed environments. For example, in an automatic monitoring system [1], event recognition is the key to detect anomalies for quick response. Currently, low-level sensing data obtained in smart spaces (e.g., smart home and smart warehousing) is the main source of information for event recognition. Considerable research has been devoted to human activity recognition (a subfield of event recognition) by exploiting different types of sensors from vision sensors like cameras to sensors that provide binary outputs (on or off), such as contact switch sensors detecting door opened or not. However, the main problem of sensor-based event recognition is that the data obtained from sensors have different degrees of uncertainty and dynamics [2,3]. This uncertainty arises for a number of reasons in a sensory network environment, such as faulty sensors, inaccurate measuring (always not 100% accurate) and “dirty” data corrupted by wireless networks due to network problems. Since the uncertainty of sensing data is inevitable, the issue comes down to the accommodation of possible existing uncertainty. Furthermore, because the state of sensors and network environments may dynamically change, the problem that the uncertainty of sensing data changes over the time should also be taken into full consideration.

Sometimes, sensing data is not sufficiently descriptive for an event. In some complex situations, the state of diverse situational items and their relationships are required for the detection of complex environmental problems within a specific context. A possible way to cope with this issue is to cooperatively apply such observed data with other contextual information. For instance, human behavior is likely to be depicted more explicitly on the basis of the circumstance, prior knowledge, etc. hence generic sensory and conceptual models are regarded as effective ways to extract and further process the contextual information for human activity recognition [4,5]. Currently, the increasing interest in contextual information has led to considerable research on the higher-level information fusion [6,7]. From the standpoint of fusion-based recognition, a context can be defined informally as a set of background conditions that have potential relevance to optimal processing, but are not primarily concerned in the system. When a context is provided, (i.e., some conditions hold), more meaningful information is available for the complex event recognition and thus the recognition accuracy is improved. Since the contextual information is usually presented in various forms, such as constraints or complementary knowledge, the diversity makes the fusion of contextual information a significant task for context-aware event recognition.

In this paper, we propose a multi-level information fusion model that explicitly fuses sensing data and useful contextual information about events. In addition, we present an approach for event recognition based on Markov logic networks where a set of pairs, i.e., (formula, weight) is exploited to describe the relationships among information items. The formulas are achieved from domain knowledge by using the information fusion model and the associated weights are learned from historical data through a statistical learning method. To tackle the problem that the relationships among information items are dynamically changing in unstable situations, we propose a dynamic weight updating algorithm on the basis of our information fusion model. The remainder of this paper is organized as follows: Section 2 mainly introduces the related work of context-aware event recognition and Section 3 provides a brief introduction of MLNs. Section 4 presents our MLNs-based approach for event recognition including the information fusion model, statistical learning method for formula weights and further detailed steps of the dynamic weight updating algorithm. The experiments and results are presented in Section 5. Finally, the paper is concluded in Section 6.

## 2. Related Work

Modeling for the representation of sensing data, contextual information, events and their relationships is a major challenge for context-aware event recognition, and the topologic structure is one of the most important features of those models. For example, Yang [8] presented a three-level model, i.e., sensor-location-action and based on this model locations are estimated from sensory readings and an action sequence is inferred from locations for activity recognition. Yurur et al. [4,5] introduced a clear description of context and a two-level context model (i.e., low-level context and high-level context) where the low-level context is the atomic relevant context and the high-level context is the composition of low-level and/or high-level contexts. Also, a tree-like model was proposed to present the relationships among sensing data, signal features and postural actions [9]. In [10], Wang et al. proposed an event-centric context model which incorporates three contexts, i.e., event temporal context, scene context and event-object interaction context, and utilized the model to recognize events in surveillance videos. Furthermore, based on descriptive models, the reasoning over uncertain information is also a tough issue for context-aware event recognition. Currently, a number of approaches for uncertain information management were proposed founded on description logics [11,12,13,14,15]. For instance, Almeida et al. [12] proposed an ontology-based approach for modeling and managing the user context with consideration of uncertainty and vagueness in contextual data. On the foundation of a tree-like conceptual model, this approach applied an inference process for obtaining a picture of smart environments. Aiming at the information retrieval, Nottelmann et al. [13] put forward the probabilistic OWL Lite subsets using the probabilistic data log. Based on Bayesian networks, some probabilistic extensions to ontology were proposed to represent and reason on uncertain information, such as PR-OWL [14] and BayesOWL [15]. In a word, those approaches we present earlier are capable of modeling context or reasoning over uncertain contextual information (with none or a weak learning process). To recognize events with different data sources, however, more generic descriptive models and enhanced inference methods with a powerful learning mechanism are required for the underlying information fusion.

Sensing data is inherently imperfect and the data uncertainty may easily arise due to: (1) erroneous or missing sensor readings and (2) the diversities of data sources or modalities. Thus the accurate recognition of events on the basis of sensing data is a challenging task. Allowing a principled manner for managing data uncertainty, probabilistic graphical models (PGMs) are advantageous and commonly used for sensor-based event recognition. The Hidden Markov model (HMM) is probably the most suitable approach for activity recognition as it can model the temporal information in the sensing data [16]. An HMM consists of a set of hidden states coupled in a stochastic Markov chain and observable states probabilistically generated by those hidden states. Generally, activities are mapped to the hidden states and sensory readings are mapped to the observable states in the HMM-based approaches for event recognition [17,18,19]. The HMM has many extensions, such as the coupled HMM [20], hierarchical HMM [21] and parallel HMM [22]. Similar to the HMM, the conditional random field (CRF) [23] is adopted to estimate the most likely sequence of hidden states (activities) based on the known sequence of observable states (sensing data). As the simplest form of the CRF, a linear-chain CRF resembles most closely the HMM but it does not assume any independency among the observable states. Unlike the HMM and CRF, a naive Bayes model assumes that all data points (e.g., sensory readings) are independently distributed, and it does not consider any temporal relationships among data points [24]. A Bayesian network (BN) is a directed acyclic graph that describes a set of variables and their conditional dependencies. For instance, in [25], a BN is exploited to represent the probabilistic relationship among abnormal events and features extracted from sensing data. In general, probabilistic graphical models mentioned above are the most well-known computation models utilized in the sensor-based activity recognition. However, a large volume of carefully labeled training data for parameter learning deters the use of those probabilistic models [17,26].

Although sensing data is able to provide essential information about occurring events, sometimes extra explicit domain knowledge is required for complex event recognition. Currently there are a number of research works in event recognition through the logic-based models, such as temporal logic [27], event calculus [28], fuzzy Logic [29] and action description logic [30]. For example, temporal-logic-based formalisms were used to specify critical sequences of low level events over the time in [27]. Since event calculus can naturally represent the relational structures of complex events, in [28], the event calculus was applied to describe the interesting background knowledge by its formal and declarative semantics. Despite of the limitation of data obtained from scenarios, most logic-based approaches are capable of facilitating the use of domain dependent knowledge and supporting the integration of heterogeneous data. These approaches, however, can hardly deal with uncertain data. To cope with uncertainty, an interval-based approach was presented through probabilistic event logic which jointly model events with respect to time intervals and their spatiotemporal relationships [31]. Markov logic networks, combining the expressivity of first order logic (FOL) and the uncertainty disposal of probabilistic graphical models, are recently applied to event recognition in maritime domain [32,33], activity recognition of daily living [34], etc. For example, Skarlatidis et al. [35] proposed a method (MLN-EC) which combines a variant of the event calculus with the probabilistic framework of MLNs. The inputs of MLN-EC are composed of simple event streams and a set of domain-dependent complex event definitions. After generating a compact knowledge base from the inputs, the method employs MLNs to perform the learning step and probabilistic inference step to recognize the interesting complex events. Assuming that information is already presented in a symbolic form, the majority of those MLNs-based methods have a very powerful and intuitive knowledge structure. Nevertheless, it is generally unclear how the raw sensing data is integrated into the logic model and fused with other contextual information. Besides, few of them address the issue that the uncertainty degree of sensing data is dynamically changing in Internet of Things environments with inherent dynamism.

## 3. Preliminaries

Markov logic networks (MLNs) are regarded as an excellent combination of the FOL and PGMs. The essential definition of MLNs is offered in this paper and more details can be found in [36]. A Markov logic network is a set of FOL formulas that are constructed recursively by atomic formulas, logical connectives, etc. Additionally, a real number is attached to each of the formulas as a weight. From the logic perspective, a weighted formula is a soft constraint so that it has a remarkable capability of inconsistent reasoning under uncertain knowledge. On the probability side, MLNs can define a discrete probability distribution on possible worlds. A world is indicated by an assignment of truth values to all atomic formulas (i.e., FOL predicates with variables or constants). If other things are equal, the world with a violation of a formula is less possible than the one satisfying this formula, rather than impossible.

A Markov logic network $M_{L}$ (i.e., the pairs of ($F_{i}$, $w_{i}$)) can define a Markov network $M_{L,C}$ along with a set of constants. The characteristic of this Markov network is: (1) $M_{L,C}$ includes a binary node for every possible grounding of atomic formulas (i.e., FOL predicates) in $M_{L}$. If the ground atom is true, the node’s value is 1, and 0 otherwise; (2) $M_{L,C}$ contains edges that connect ground atoms iff those nodes co-occur in at least one ground formula (i.e., a formula containing no variables) in $M_{L}$; (3) $M_{L,C}$ assigns a feature for every grounding of formulas in $M_{L}$. If the ground formula is true, the feature’s value is 1, and 0 otherwise. Besides, the feature’s weight is $w_{i}$ that is associated with $F_{i}$.

The joint probability distribution of a possible world *x* corresponding to a specific grounding of $M_{L,C}$ is calculated as follows:

(1) $$P\left(X=x\right)=\frac{1}{Z}\exp\left(\underset{i}{\sum }w_{i}n_{i}\left(x\right)\right)$$

where $Z=\sum_{x^{\prime }\in \chi }\exp\left(\sum_{i}w_{i}n_{i}\left(x^{\prime }\right)\right)$ denotes a normalizing factor for proper probability estimation, χ is the set of all possible worlds and $n_{i}\left(x\right)$ denotes the total number of true groundings of $F_{i}$ in *x*. Similar to Equation (1), given two sets of atoms *X* and *Y*, the conditional likelihood of *Y* under *X* is:

(2) $$P_{\omega }\left(y|x\right)=\frac{1}{Z_{x}}\exp\left(\underset{i\in F_{Y}}{\sum }w_{i}n_{i}\left(x,y\right)\right)$$

where $F_{Y}$ is a set of all FOL formulas whose grounding is involved in at least one atomic formula in Y and $n_{i}\left(x,y\right)$ denotes the total number of true groundings of $F_{i}$ in $F_{Y}$. In this paper, we take the atomic formulas in terms of sensing data and known contextual information as evidence atoms, meanwhile take the atomic formulas related to events as query atoms. The relationships among query atoms and evidence atoms are modeled as conditional dependencies.

## 4. Our MLNs-Based Approach for Event Recognition

### 4.1. Multi-Level Information Fusion Model

**Figure 1:**
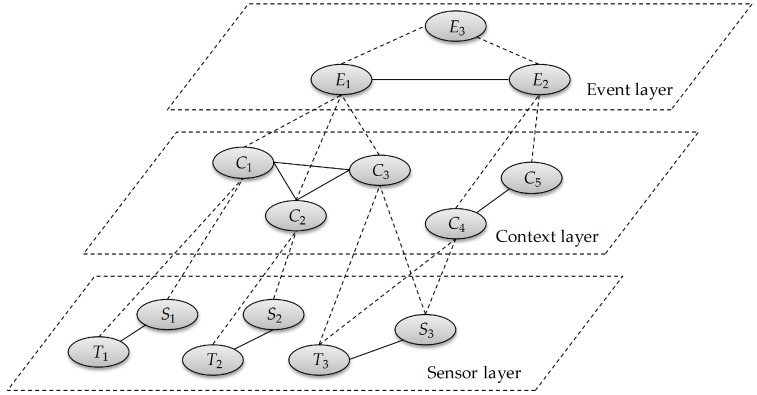
Three-level information fusion model for event recognition.

Inspired by those existing approaches, we define a more generic information fusion model with three layers (i.e., sensor layer, context layer and event layer) for event recognition, as shown in Figure 1. For the convenience of illustration, information here is presented in the form of FOL predicates. The nodes in different layers imply various predicates and edges denote the direct (by solid lines) or indirect (by dashed lines) relationships between these predicates. Nodes in the sensor layer are categorized into two types (sensor nodes (*S_n_*) and trigger nodes (*T_n_*)) which respectively identify heterogeneous sensors (e.g., ID and location) and describe their trigger conditions that decide when and how those sensors are activated to reveal the interesting contextual information. To represent the profound information hidden in sensing data, some labels are provided to annotate the discrete data or continuous data when crossing specific thresholds. For example, labels “*low*”, “*middle*” and “*high*” are respectively employed to identify that the output value of a temperature sensor is lower than 25 °C, between 25 and 40 °C or higher than 40 °C. In other words, if the output of a temperature sensor is 34 °C, the truth value of its trigger predicate grounded by “*middle*” is 1.

The nodes in the context layer refer to the predicates about contextual information. Since the information corresponding to sensor nodes maybe not sufficient to reflect the occurrence of complex events, the extra contextual information should be further considered in the context layer. For example, the recognition of an explosion event with respect to a couple of compound attributes including temperature, sound and luminance is obviously more accurate than the one recognized with merely one of these attributes. Also, the domain knowledge given by experts, e.g., temperature constraints of chambers, is essential to identify anomalies in a logistic storage. The main goal of the context layer is to fuse information acquired from underlying sensing data, domain knowledge, etc. Events here are mapped to event nodes in the event layer. They are divided into two types: simple events (e.g., *E*_1_ and *E*_2_, the most obvious and basic status changes captured by sensing data) and complex events (e.g., *E*_3_, composed by simple events or additional contextual information).

**Figure 2:**
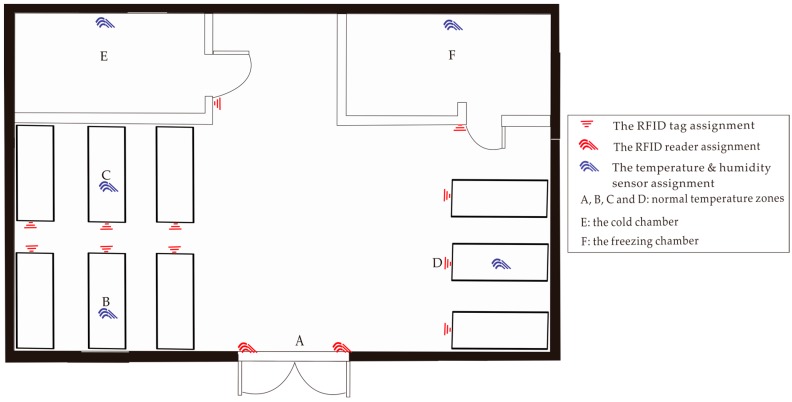
The layout of the storage with deployed sensors.

As shown in Figure 2, we adopt throughout the paper an exemplary scenario of the logistic storage which consists of four normal temperature zones (A, B, C and D), a cold chamber (E) and a freezing chamber (F). The logistic storage is equipped with different sensing devices to obtain the information implying the event occurrence for surveillance and control. In a logistic storage, products are usually packaged in boxes, containers or pallets for some reasons such as protection or portability. For simplicity, storage units mentioned above are entirely called storage objects and each of them is assumed to be attached with an RFID tag. The RFID tags are used to extract the information of storage objects for RFID readers. For example, if a RFID tag is read by the RFID reader set beside the door, the system is able to get a record on goods-in or goods-out of the related objects. The RFID tag is also useful to identify goods shelves and locations if RFID tags are bounded to them. The temperature and humidity values of zones and chambers are captured by temperature & humidity sensors also deployed around this storage. Related to [37], storage objects have constraints on their movement plan (e.g., moving through planned locations) and attributes (e.g., temperature and humidity). When a state alteration of a tagged storage object is probed by a sensor, an event occurs. The state of a storage object is one of those below and the basic events of storage objects are shown in Table 1:

- Normal (*n*): a storage object does not validate against its constraints on the movement plan and attributes.
- Violation of movement plan (*m*): a storage object is deviated from its movement plan to an unscheduled location.
  *m*1: Violating the movement plan in the goods-in phase;
  *m*2: Violating the movement plan in the inventorying phase;
  *m*3: Violating the movement plan in the goods-out phase.
- Violation of attribute constraints (*a*): this state is activated when any property value of a storage object is beyond the range of its attribute constraints.
  *a*1: Violating the temperature attributes;
  *a*2: Violating the humidity attributes.

**Figure 3:**
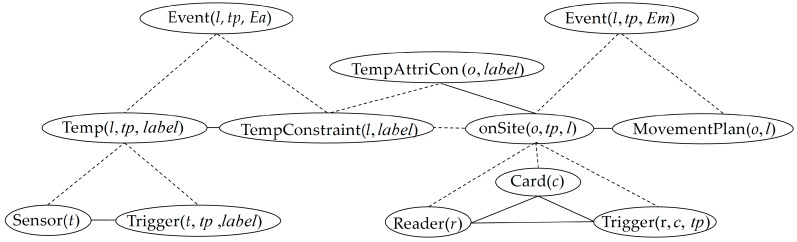
A part of the information fusion model referring to formulas #1, #3, #4, #6 and #8–#10 in Table 2.

Based on the description of events and domain knowledge provided by experts, we build the specific information fusion model for this scenario and the primary part is shown in Figure 3. On the basis of the model, we further establish a knowledge base depicted by FOL whose corresponding rules are partly shown in Table 2. Note that items in a predicate with double quotes denote constants while variables are without quotes. The first three rules indicate that sensors (temperature sensor *t*, humidity sensor *h* and RFID reader *r*) deployed on location *l* are triggered to access the contextual information including the environment temperature and humidity with predefined labels “*high*” and “*low*”(#1 and #2), and the location of object *o* at the time point *tp* (#3). The temperature and humidity constraints of locations are set consistent with the constraints of storage objects at those locations (#4 and #5). The description of five typical events is shaped by rules #6–#10. When the temperature or humidity of location *l* violates their corresponding constraints, event “*Ea*1” or “*Ea*2” occurs separately (#6 and #7). If an object moves to an unplanned location at time point *pt*, event “*Em*1”, “*Em*2” or “*Em*3” happens. Based on the established information fusion model, we build the Markov network aiming at the given scenario.

### 4.2. Statistical Learning Method for Formula Weights

Formula weights are the key features of MLNs to handle uncertainty. Instead of set manually, formula weights in this paper are learned from training data. We divide training data into two types, i.e., evidence atoms and query atoms, and then apply the diagonal Newton method [38,39] to approximately optimize the conditional likelihood shown as Equation (2).

Newton’ method, sometimes known as Newton’s iteration, is a root-finding algorithm in optimization. From initial guess root $X_{0}$, it constructs a sequence $X_{n}$ that converges towards some $X^{*}$ satisfying the minimum or maximum of an objective function. As for a multivariate objective function, the iterative formula is shown as Equation (3), where g is the gradient of the objective function and $H^{-1}$ is the inverse of the Hessian matrix:

(3) $$X_{t+1}=X_{t}-H^{-1}g$$

On account of the large quantity of weights, we utilize the diagonal Newton method that exploits the inverse of a diagonalized Hessian instead of the inverse of Hessian. For simplicity, we calculate the partial derivative of the logarithm of Equation (2) (i.e., conditional log-likelihood) with respect to a weight and then obtain:

(4) $$\frac{\partial }{\partial \omega_{i}}\log P_{\omega }\left(y|x\right)=n_{i}\left(x,y\right)-E_{\omega }\left[n_{i}\left(x,y\right)\right].$$

where $n_{i}\left(x,y\right)$ is the total number of the true ground formulas according to the training data and $E_{\omega }\left[n_{i}\left(x,y\right)\right]$ is the expectation of $n_{i}\left(x,y\right)$.

Next, the Hessian of the conditional log-likelihood can be denoted by the negative covariance matrix as follows:

(5) $$\frac{\partial^{2}\left(\log P_{\omega }\left(y|x\right)\right)}{\partial \omega_{i}\partial \omega_{j}}\log P_{\omega }\left(y|x\right)=E_{\omega }\left[n_{i}\right]E_{\omega }\left[n_{j}\right]-E_{\omega }\left[n_{i}n_{j}\right],$$

where $n_{i}\left(x,y\right)$ is abbreviated by $n_{i}$. Because the accurate calculation of the gradient and Hessian is impractical, they can be evaluated with samples from MC-SAT [40]. According to Equations (3) to (5), we use the search direction of diagonalized Newton and then take the iteration formula in each iteration step:

(6) $$\omega_{i}=\omega_{i}-\alpha \frac{n_{i}-E_{\omega }\left[n_{i}\right]}{E_{\omega }\left[n_{i}^{2}\right]-{{(E}_{\omega }\left[n_{i}\right])}^{2}}$$

where α is a step size. There are numbers of ways to calculate α, including keeping it fixed. Let *d* denote the search direction and *H* denote the Hessian matrix, we calculate α by Equation (7):

(7) $$\alpha =\frac{d^{T}g}{d^{T}Hd+\lambda d^{T}d}$$

where nonzero λ is employed for the limitation of the step size to some range for good quadratic approximation. After each iteration, λ is adjusted according to how well the approximation matches the objective function. Let $\Delta f_{actual}$ and $\Delta f_{pred}$ denote the actual change and predicated change in the function value respectively, we adopt a standard method to adjust λ as follows:

(8) $$\lambda_{t+1}=\{\begin{array}{cc} \lambda_{t}/2 & \Delta f_{actual}/\Delta f_{pred}>0.75\\ \lambda_{t} & 0.25\leq \Delta f_{actual}/\Delta f_{pred}\leq 0.75\\ 4\lambda_{t} & \Delta f_{actual}/\Delta f_{pred}<0.25 \end{array}.$$

According to Taylor series of the objective function, we compute $\Delta f_{pred}$ by Equation (9):

(9) $$\Delta f_{pred}=d_{t-1}^{T}g_{t-1}+\left(d_{t-1}^{T}H_{t-1}d_{t-1}\right)/2.$$

Since the actual change in conditional log-likelihood can not be calculated efficiently, we proximate it through $\Delta f_{actual}=d_{t-1}^{T}g_{t}$. As the conditional log-likelihood is convex, the product of $\Delta f_{actual}$ is a lower bound of the increment in its actual value. Note that if this product is negative, the corresponding iteration step has to be interrupted and redone after the readjustment of λ. The iteration will not come to an end until the preset stopping criterion (e.g., max iteration time) is satisfied, and we take the average weight from all iteration steps as the final result.

### 4.3. Dynamic Weight Updating Algorithm

In this study, events are recognized by inference based on the MLNs with formula weights learned from training data. Compared with the testing data for event recognition, the training data is commonly collected over a long time span. Therefore, it is difficult to be aware of the short-term data uncertainty for weight learning. Moreover, an inference may no longer hold due to the dynamics (modification or invalidation) of temporal data. As a consequence, the reason strategy plays an important role in accommodating data dynamics. Here, we propose a dynamic weight updating algorithm on the foundation of the three-level information fusion model mentioned in Section 4.1. The goal of this algorithm is to update the unsuitable weights during event recognition. Assume that events are recognized in chronological order and the total number of the incorrectly recognized events is recorded. When this number exceeds a given threshold, all the formula weights associated to the incorrectly recognized events are considered to be updated by Algorithm 1. To illustrate the proposed algorithm, consider two definitions:

**Definition 1** (A factor sensor of an event)**.** If a sensor node is connected directly or indirectly with an event node in the information fusion model, then it is called as the factor sensor of the event.

**Definition 2** (A relevant rule of a factor sensor)**.** If an inference rule in the knowledge base includes a predicate according to a sensor, then it is called as the relevant rule of the factor sensor.

**Algorithm 1. Dynamic Weight Updating Algorithm****Input:**  A Markov logic network $M_{L,C}$, the number of time slices *N*, an event set consisting of incorrectly recognized events $S_{E}$, a dataset consisting of testing data used before the current timestamp, $TestingData_{u}$.**Output:** updated $M_{L^{\prime },C}$**Step 1:**
$S_{FS}=\left(s_{1},\;s_{2},\ldots s_{k}\right)$ ← the factor sensors in terms of events in $S_{E}$.**Step 2:**
$S_{r}=\left(r_{1},\;r_{2},\ldots r_{l}\right)$ and $S_{w}=\left(\omega_{1},\omega_{2},\ldots \omega_{l}\right)$ ← the relevant rules and weights with respect to factor sensors in $S_{\mathrm{FS}}$.**Step 3:**
**if** the size of ($TestingData_{u})\geq N$, then  $TrainingData_{u}$ ← the data on the *N* nearest time slices in $TestingData_{u}$.  **else**
${\mathrm{TraningData}}_{u}$ ← $TestingData_{u}$.**Step 4:** achieve $S_{\omega }^{\prime }\left(\omega_{1}^{\prime },\;\omega_{2}^{\prime },\;\ldots \;\omega_{l}^{\prime }\right)$ from $TrainingData_{u}$ by the statistical learning method in Section 4.2.**Step 5:** obtain $M_{L^{\prime },C}$ with updated $S_{\omega }^{\prime }\left(\omega_{1}^{\prime },\;\omega_{2}^{\prime },\;\ldots \;\omega_{l}^{\prime }\right)$.**Return:**
$M_{L^{\prime },C}$

The inputs of the proposed algorithm are a Markov logic network $M_{L,C}$, the number of time slices *N*, an event set consisting of incorrectly recognized events $S_{E}$, and a data set consisting of testing data used before weight updating, $TestingData_{u}$. The algorithm first constructs a sensor set $S_{\mathrm{FS}}$ whose elements are factor sensors of all events in $S_{E}$. In the next step, all relevant rules of factor sensors in $S_{\mathrm{FS}}$ are selected to construct a rule set $S_{r}=\left(r_{1},r_{2},\ldots r_{l}\right)$ and its corresponding weight set is $S_{w}=\left(\omega_{1},\omega_{2},\ldots \omega_{l}\right)$. Note that rules in the knowledge base can be encoded as a FOL formula. In step 3, the data on the *N* nearest time slices in $TestingData_{u}$ are extracted into a new training data set $TrainingData_{u}$ for weight updating. Note that if the number of the time slices of $TestingData_{u}$ is smaller than *N*, all the data in $TestingData_{u}$ are put into $TrainingData_{u}$. In step 4, it achieves the new weights of the relevant rules by the statistical learning method presented in Section 4.2. Finally, the algorithm obtains the updated $M_{L^{\prime },C}$.

## 5. Experiments

We used two exemplary scenarios to demonstrate the effectiveness of the proposed information fusion model, statistical learning method for weights and dynamic weight updating algorithm. The first scenario is the activity recognition of a young man who lives alone in an apartment with three rooms. The second one is the event detection in the complex logistic storage where the dynamic uncertainty of data arises due to simulated dynamic situations. The data collected from each scenario is split into two sets (i.e., training data and testing data). We first obtain the domain-specific knowledge base by the information fusion model. Then through the statistical learning method, the weights associated to rules in knowledge base are learned from training data. In the end, we recognize interesting events (activities) hidden in testing data by an open source inference engine Tuffy [41] designed for MLNs. The values of some parameters are discussed below. In weight learning phase, the default values for the initial weight, λ and max iteration time are set 1, 1 and 100 respectively. The performance of the proposed method is evaluated by four widely used scales, i.e., accuracy, precision, recall and F-measure.

### 5.1. Datasets

One of the two datasets used in our study is extracted from the publicly available Ubicomp dataset [42]. It is collected by a wireless sensor network (deployed in scenario 1) which consists of 14 nodes. A signal can be sent by a node according to the threshold excess of the analog input or the state change of the digital input. Each of these nodes is equipped with a sensor, such as reed switch sensors used to measure the state of cupboards and doors, contact sensors for the detection of object movements and float sensors to probe the flushing of toilets. The data is presented as a list of tuples (*timestamp*, *ID*) for 28 days. Totally, there are 2120 simple sensory readings and 245 activity instances annotated by the following 7 types of labels: “*Take shower*”, “*Use toilet*”, “*Leave house*”, “*Get drink*”, “*Prepare dinner*”, “*Prepare breakfast*” and “*Go to bed*”.

Due to the deficiency of appropriate public datasets for the evaluation of event recognition in high dynamic and complex environments, the simulated data is generated from the synthetic logistic storage scenario mentioned above. Compared with scenario 1, it is involved in more complex sensing devices (e.g., RFID), contextual information (e.g., business logic) and dynamic situations (e.g., device fault and packet loss of networks). To comprehensively study the dynamic uncertainty of sensing data, we assume that uncertain data is categorized into two types as the consequence of dynamic situations: (1) the sensing data that should be sent from sensors but incorrectly obtained in the system and (2) the sensing data that should not be sent from sensors but unexpectedly received in the system. We utilize a parameter, dynamic rate ϖ, to indicate the degree of dynamics, which is defined as the ratio of the total number of time slices of uncertain data to the total number of time slices in the data group. With different values of the dynamic rate ϖ, the six kinds of events mentioned in Table 1 and other anomalies caused by uncertain data occur continually, and then the corresponding sensing data is recorded into the simulated dataset. Moreover, to evaluate the effect of the events’ frequency on the performance of the dynamic weight updating algorithm, events related to a storage object are classed into two categories, i.e., high frequency event (with occurrence frequency >75%) and low frequency event (with occurrence frequency <25%). For each type of the two event categories, 5 groups of data are generated with the same dynamic rate and each group has about 1200 time slices (time slice length Δt=60 s).

### 5.2. Results and Analysis

The goal of the study in scenario 1 is to validate the effectiveness of the proposed information fusion model and statistical learning method for formula weights. We utilize the Ubicomp dataset with a “leave-one-out” approach in which sensing data of one full day is used as testing data and the remaining is utilized for training. Considering the layout of the house and deployment of sensors, we establish a corresponding information fusion model and learn all weights from training data for inference. We traverse all the days and calculate the average accuracy of event recognition. Moreover, to demonstrate that our method performs as well as typical probabilistic models for activity recognition, we compare it with the HMM and CRF. Table 3 provides a comparison of performance with respect to accuracy in the Ubicomp dataset. All the three approaches are competitive in accuracy, and our MLNs-based method does slightly better than the HMM.

To validate the advantage of the dynamic weight updating algorithm, we further implement the experiment on the simulated data with 5-fold cross validation which takes one group of data as testing data and trains four groups of data for weight learning. According to the domain knowledge of scenario 2 provided by experts, we build the specific information model and knowledge base mentioned in Section 4.1. After the initial weight learning, we recognize events in two ways, (1) inference by using Tuffy along with the proposed dynamic weight updating algorithm (UMLNs) and (2) inference just by Tuffy (i.e., the original MLNs-based method or MLNs).

**Figure 4:**
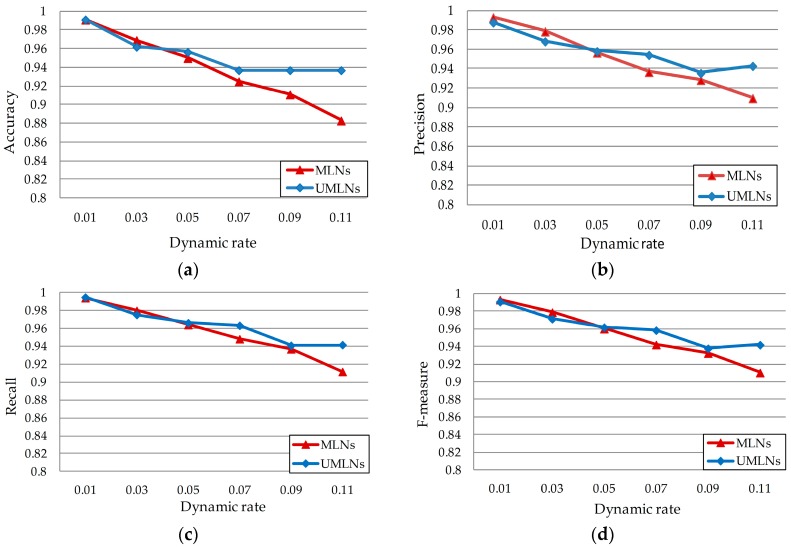
Dynamic event recognition for high frequency events with respect to four scales, (**a**) Accuracy; (**b**) Precision; (**c**) Recall; (**d**) F-measure.

Figure 4a–d shows the effect of the dynamic weight updating algorithm when high frequency events occur in dynamic environments. When ϖ<0.05, UMLNs perform slightly worse than the MLNs method. The reason is that once the UMLNs capture the exceptional event patterns, deriving from uncertain data, our approach can recognize those exceptional events more correctly, while the acquisition of exceptional event patterns may have negative impacts on the recognition of normal events. Therefore, the arising of false recognition for normal events can be treated as an essential updating cost. With the increase of dynamic rate, the advantages of UMLNs can remedy the limitation of this cost. When ϖ=0.05, UMLNs achieve equivalent performance as the MLNs method in aspects of precision, recall and F-measure, and even a superior result in accuracy. Additionally, when ϖ>0.05, UMLNs outperform the MLNs in terms of all the four metrics.

**Figure 5:**
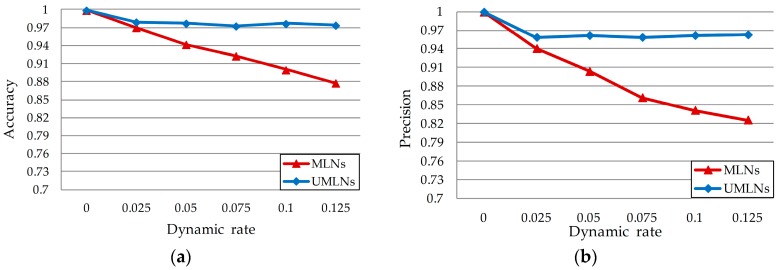

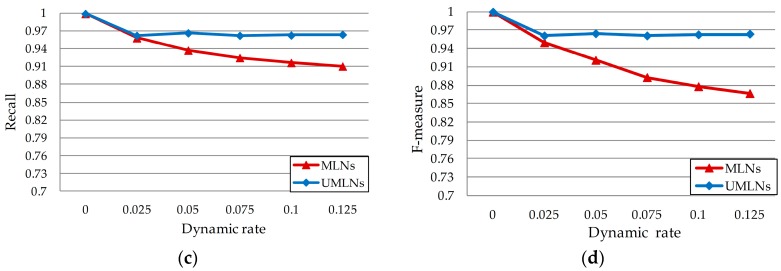
Dynamic event recognition for low frequency events with respect to four scales, (**a**) Accuracy; (**b**) Precision; (**c**) Recall; (**d**) F-measure.

As illustrated for low frequency events in Figure 5a–d, when ϖ=0.025, UMLNs obviously achieve preferable performance in terms of precision, and identical performance in accuracy, recall and F-measure with the MLNs. When ϖ>0.025, UMLNs transcend the MLNs method in four aspects overall. The possible cause is that our algorithm adapts effectively to the data dynamic once the dynamic rate reaches a certain level correlated to the events’ frequency. Along with Figure 4 and Figure 5, it further indicates that, compared with high frequency events, dynamic situations related to low frequency events are more adaptable to our approach.

## 6. Conclusions

In this paper, we investigate the problem of event recognition through fusing uncertain sensing data and contextual information in dynamic environments. Although there exist a number of event recognition approaches, few of them address the issues of fusing uncertain sensing data with contextual information and handing data dynamics. The main contribution of our work consists of three parts: (1) a newly proposed multi-level information fusion model; (2) a clearly presented approach for event recognition on the basis of MLNs, which obtains formulas by using the information fusion model and learns formula weights through the diagonal Newton’ method; (3) the dynamic weight updating algorithm used for handing data dynamics. Implemented on two datasets, the experiments show that our approach provides an effective way to recognize events through the fusion of uncertain data and contextual information, and outperforms the original MLNs-based method especially in dynamic environments.

## Figures and Tables

**Table 1:** Typical events of a storage object.

Event	State Transition		Description
*En*	*N* → *n*		Transition between normal states
*Em*1	*n* → *m*	*n* → *m*1	Violating the movement plan in the goods-in phase
*Em*2		*n* → *m*2	Violating the movement plan in the inventorying phase
*Em*3		*n* → *m*3	Violating the movement plan in the goods-out phase
*Ea*1	*n* → *a*	*n* → *a*1	Violating constraints on the temperature attribute
*Ea*2		*n* → *a*2	Violating constraints on the humidity attribute

**Table 2:** A Part of the knowledge base for event recognition in the scenario of logistic storage in FOL.

#	Rules
1	isLocated (t, l)∧Trigger (t, tp, “high”) → Temp (l, tp, “high”)
2	isLocated (h, l)∧Trigger (h, tp, “low”) → Humidity (l, tp, “low”)
3	isLocated (r, l)∧isAttached (o, c)∧Trigger (r, c, tp) → onSite (o, tp, l)
4	onSite (o, tp, l)∧TempAttriCon (o, label) → TempConstraint (l, label)
5	onSite (o, tp, l)∧HumidityAttriCon (o, label) → HumidityConstraint (l, label)
6	Temp (l, tp, “high”)∧TempConstraint (l, “high”) → Event (l, tp, “Ea1”)
7	Humidity (l, tp, “low”)∧HumidityConstraint (l, “low”) → Event (l, tp, “Ea2”)
8	onSite (o, tp, l)∧(l=“goodsInSite”)∧┐MovementPlan (o, “goodsInSite”) → Event (l, tp, “Em1”)
9	onSite (o, tp, l)∧(l=“storageSite”)∧┐MovementPlan (o,“storageSite”) → Event (l, tp, “Em2”)
10	onSite (o, tp, l)∧(l=“goodsOutSite”)∧┐MovementPlan (o, “goodsOutSite”) → Event (l, tp, “Em3”)

**Table 3:** The performance of our MLNs-based method, HMM and CRF.

	HMM	MLNs	CRF
Accuracy	94.5%	95.5%	95.6%
